# Increased Virulence of an Epidemic Strain of *Mycobacterium massiliense* in Mice

**DOI:** 10.1371/journal.pone.0024726

**Published:** 2011-09-12

**Authors:** Shaobin Shang, Sara Gibbs, Marcela Henao-Tamayo, Crystal A. Shanley, Gerald McDonnell, Rafael Silva Duarte, Diane J. Ordway, Mary Jackson

**Affiliations:** 1 Mycobacteria Research Laboratories, Department of Microbiology, Immunology and Pathology, Colorado State University, Fort Collins, Colorado, United States of America; 2 STERIS Limited, Basingstoke, England; 3 Instituto de Microbiologia, Universidade Federal do Rio de Janeiro, Rio de Janeiro, Brazil; University of Edinburgh, United Kingdom

## Abstract

**Background:**

Chronic pulmonary disease and skin/soft tissue infections due to non-tuberculous mycobacteria (NTM) of the *Mycobacterium chelonae-abscessus-massiliense* group is an emerging health problem worldwide. Moreover, the cure rate for the infections this group causes is low despite aggressive treatment. Post-surgical outbreaks that reached epidemic proportions in Brazil recently were caused by *M. massiliense* isolates resistant to high-level disinfection with glutaraldehyde (GTA). Understanding the differences in the virulence and host immune responses induced by NTM differing in their sensitivity to disinfectants, and therefore their relative threat of causing outbreaks in hospitals, is an important issue.

**Methodology/Principal Finding:**

We compared the replication and survival inside macrophages of a GTA-susceptible reference *Mycobacterium massiliense* clinical isolate CIP 108297 and an epidemic strain from Brazil, CRM-0019, and characterized the immune responses of IFNγ knockout mice exposed to a high dose aerosol with these two isolates. CRM-0019 replicated more efficiently than CIP 108297 inside mouse bone marrow macrophages. Moreover, the animals infected with CRM-0019 showed a progressive lung infection characterized by a delayed influx of CD4+ and CD8+ T cells, culminating in extensive lung consolidation and demonstrated increased numbers of pulmonary CD4+ Foxp3+ regulatory T cells compared to those infected with the reference strain. Immunosuppressive activity of regulatory T cells may contribute to the progression and worsening of NTM disease by preventing the induction of specific protective immune responses.

**Conclusions/Significance:**

These results provide the first direct evidence of the increased virulence in macrophages and mice and pathogenicity *in vivo* of the Brazilian epidemic isolate and the first observation that NTM infections can be associated with variable levels of regulatory T cells which may impact on their virulence and ability to persist in the host.

## Introduction

Non-tuberculous mycobacteria (NTM) are ubiquitous in the environment and are responsible for colonization, infection and pseudo-outbreaks in health care settings throughout the world. Most NTM nosocomial outbreaks caused by rapidly growing mycobacteria (RGM) have involved the species *M. abscessus*, *M. chelonae*, *M. fortuitum* and two recently recognized *Mycobacterium* species closely related to *M. abscessus*: *M. massiliense* and *M. bolletii* [1], [2]. These pathogens can cause a wide range of clinical diseases including skin and soft tissue infections, pulmonary, central nervous system and disseminated infections [3], [4], [5], [6], [7], [8], [9], [10], [11], [12], [13], [14]. While disseminated infections caused by RGM essentially occur in immunodeficient patients and pulmonary infections are widely thought to be opportunistic and to occur in patients with structural lung disease (e.g., chronic obstructive pulmonary disease, bronchiectasis, cystic fibrosis), numerous cases of skin and soft tissue infections have been reported in otherwise immunocompetent patients, caused by contaminated materials and invasive procedures involving catheters, non-sterile surgical procedures, injections and implantations of foreign bodies. Recent published reports [15], [16] and outbreaks in Brazil [13], [17] suggest that the prevalence of infections caused by these microorganisms is increasing and that infections due to RGM are particularly problematic due to their ubiquitous presence in hospitals' water sources and the difficulty of treating some of the infections they cause.

Following infection, very little is known about the host immune response and what enables NTM to persist in host tissues. While some NTM species, such as *M. fortuitum* and *M. chelonae*, can be controlled by innate immunity [18], [19], others like *M. abscessus*, require adaptive immunity for efficient control of the infection [19], [20]. Our studies support the early occurrence of phagocytosis of *M. abscessus* by antigen-presenting cells such as alveolar macrophages and dendritic cells [20], [21]. Autocrine and paracrine activation of dendritic cells by IL-12 p40 and migration of the infected dendritic cells to the regional mediastinal lymph nodes and subsequent mycobacterial antigen presentation to naive T cells results in differentiation and further activation of effector T cells. The accumulation in the lung of protective T cells capable of expressing IFNγ and TNFα are crucial for phagocyte activation and intracellular killing [19], [22]. The development of a pulmonary granuloma is orchestrated by chemokines and cytokines produced by local cells causing a continuous recruitment of lymphocytes, macrophages, dendritic cells and granulocytes to the lung. Control of the infection relies on this granulomatous response, which is an organized cellular network acting to restrain NTM growth and limit dissemination. In the context of *M. tuberculosis* persistence and chronic disease, it has been shown that CD4+Foxp3+ regulatory T cells are important for regulation of immunopathology [23], [24], [25]. The induction of CD4+Foxp3+ regulatory T cells has never been reported in the case of *Mycobacterium chelonae-abscessus-massiliense* group persistence and we propose it is one of the strategies adopted by these microorganisms to enhance their persistence in the human host. The immunosuppressive activity of regulatory T cells may contribute to the progression and worsening of clinical disease caused by NTMs by preventing the induction of specific protective immune responses required to kill the bacterium.

The most important outbreaks – reaching epidemic proportions - of nosocomial *M. massiliense* infections ever reported occurred between 2004 and 2007 in Brazil. Interestingly, these outbreaks seem to have been caused by one single strain of *M. massiliense*, named BRA100 [12], [13], [17], which displays high level resistance to glutaraldehyde (GTA), the chemical used for disinfection of surgical instruments in all of the hospital sites that had confirmed cases. While the resistance of this particular strain to high-level disinfection has been advanced as the most likely reason for its selection and dissemination across the country [12], [13], [17], the unusual size of the outbreaks also suggests that BRA100 may represent a particularly invasive and pathogenic strain of *M. massiliense*. Such variability in pathogenicity among *M. tuberculosis* clinical isolates has been well documented [23], [26], [27], [28]. The potential increase in pathogenicity of the epidemic BRA100 isolates is further supported by our recent findings that one of the mechanisms through which RGM species develop high-level resistance to GTA involves a remodeling of the composition and/or organization of their cell surface susceptible of affecting their intracellular survival and interactions with the host [29].

We undertook this study with the goal of comparing the *M. massiliense* BRA100 epidemic isolate from Brazil to the glutaraldehyde-susceptible reference strain, *M. massiliense* CIP 108297, in their abilities to replicate inside macrophages and to infect, replicate, persist, induce organ pathology and immune responses in mice.

## Results

### Morphological traits, disinfectant resistance and in vitro growth characteristics of the *M. massiliense* isolates used in this study

*M. massiliense* CIP 108297, the type strain used in this study, was isolated from the sputum and the bronchoalveolar fluid of a patient with hemoptoic pneumonia in France in 2004 [2]. CRM-0019 is a BRA100 *M. massiliense* isolate from Brazil. It was responsible for a nosocomial infection following hepatic tumor resection by videolaparoscopy, the supposed index case of the epidemic in the state of Rio de Janeiro [13]. The female patient was 45 years old and presented the initial symptoms 90 days after the videosurgery. She was submitted to clarithromycin-based antimicrobial therapy and two additional laparotomies for granuloma resections, resulting in complete recovery. *M. massiliense* CIP 108297 and CRM-0019 display different pulse field gel electrophoresis (PFGE) patterns confirming their lack of genetic relationship [13]. The species assignment of both isolates was confirmed by partial sequencing of their *hsp65* and *rpoB* genes (view Information S1).

The plating of CIP 108297 yielded uniform colonies intermediate between rough and smooth, as described earlier [2]. Plating of CRM-0019 onto 7H11-OADC agar, in contrast, yielded a mixed population of nonphotochromogenic rough and smooth colonies. Suggestive of genetic instability, the culturing and re-plating of individual rough and smooth CFUs led to colonies with mixed phenotypes. Comparison of the growth rate of the two strains in 7H9-OADC broth at 30°C revealed only a marginal delay in growth associated to the BRA100 isolate during exponential phase (view Information S1). Their growth rate on 7H11-OADC agar plates was comparable. Suspension susceptibility tests confirmed the high level resistance of the BRA100 isolate, CRM-0019, to 2.2% GTA (view Information S1). Smooth colonies of CRM-0019 displayed the same level of resistance as the rough ones.

### Growth of *M. massiliense* strains in mouse bone marrow derived macrophages (BMDM)

**Figure 1:**
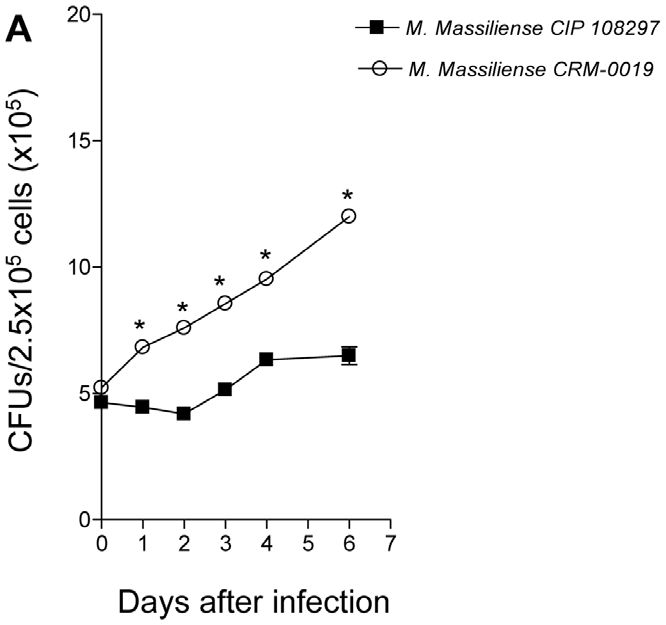
Increased intracellular replication of the Brazilian outbreak strain *M. massiliense* CRM-0019 in mouse bone marrow macrophages. Intracellular growth of *M. massiliense* reference strain CIP 108297 and *M. massiliense* outbreak strain CRM-0019 in BMDM. BMDM were infected with *M. massiliense* strains at a MOI of 5, and the numbers of intracellular bacteria were determined using the bacterial colony count method (CFU) immediately after 2 hours of infection or at 1, 2, 3, 4, 5, 6 and 7 days after infection. Values shown are the mean ± SD from two independent experiments. Growth of the *M. massiliense* outbreak strain CRM-0019 was significantly higher than the other isolate (*, *P*<0.05).

We investigated the capacity of *M. massiliense* reference strain CIP 108297 and the *M. massiliense* outbreak strain CRM-0019 to grow in BMDM to evaluate their virulence. The growth of each isolate was determined by the method of plating to measure colony-forming units (CFU; Figure 1). The *M. massiliense* outbreak strain CRM-0019 compared to the reference strain CIP 108297 demonstrated a significantly increased ability to replicate in BMDM after 24 hours of infection and steadily increased peaking at 7 days of infection. The reference strain CIP 108297 demonstrated nominal bacterial replication for 48 hours of infection and thereafter began to steadily increase in bacterial numbers in BMDM. Lastly, we evaluated the BMDM cellular viability the percentage of propidium iodide positive dead cells induced by the *M. massiliense* reference strain CIP 108297 and the *M. massiliense* outbreak strain CRM-0019 was below 6.0% for the BMDM (data not shown).

### Bacterial loads in GKO mice

We chose a pulmonary infection model using interferon-gamma (IFN-γ) gene disrupted mice (GKO) to compare the two *M. massiliense* strains *in vivo* for several reasons. Firstly, the reference strain CIP 108297 was isolated from a patient with pulmonary infection. Since our goal was to compare the pathogenicity of a BRA100 isolate from Brazil to that of CIP 108297, we wanted to ensure that the reference isolate was placed under conditions where it would perform best in terms of establishing an infection. Importantly, BRA100 isolates have been recovered both from surgery patients with skin and soft tissue infections and from patients with pulmonary infections not associated with the post-surgical epidemic [30] indicating that they are pathogenic in pulmonary infections as well. The second reason has to do with the fact that the GKO model more closely reflects an RGM infection which would occur in immunocompromised patients, a population particularly at risk for NTM infections [15]. Clinical observations and/or genetic studies in humans actually corroborate many of the findings in animals in that those with cell-mediated immunodeficiency, genetic defects in IFNγ-interleukin-12 (IL-12) axis, and those individuals on TNFα blockers are at increased risk for soft tissue infections and pulmonary NTM infections, including the RGM [21]. In addition, it has been shown that individuals infected with the human immunodeficiency virus show reduced T cell IFNγ production and TH1 immunosuppression by CD4+Foxp3+ regulatory T cell [31], [32]. Lastly, the choice of using GKO mice instead of C57BL/6 ones owes to the reported difficulty of infecting through the aerosol route wild-type C57BL/6 mice that eliminate the bacteria very rapidly, whereas GKO mice aerosolized with about 1,000 CFU develop a successful infection [33].

**Figure 2:**
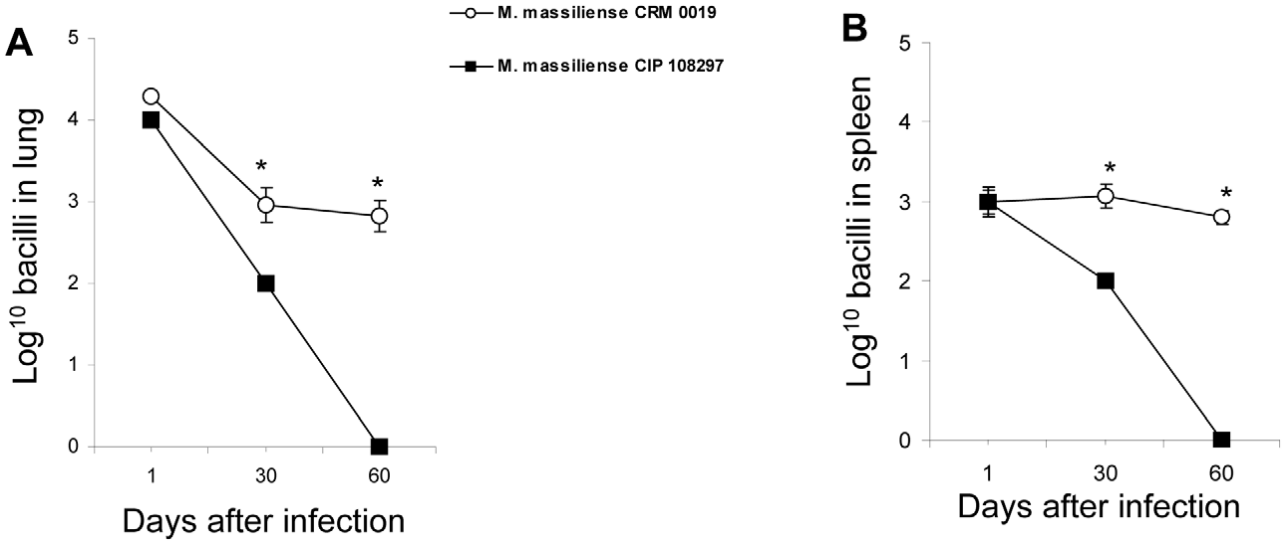
Increased bacteria in the lungs and spleens of the Brazilian epidemic strain *M. massiliense* CRM-0019 infected IFNγ-KO mice. Bacterial counts in the lungs (A) and spleens (B), on days 1, 30 and 60 from IFNγ-KO mice infected with a high dose aerosol of the epidemic strain *M. massiliense* CRM-0019 (open circle) and *M. massiliense* CIP 108297 (solid square) were compared. Results are expressed as the average (n = 5) of the bacterial load in each group expressed as Log10CFU, ± standard error mean (SEM). Student *t*-test, *p<0.050.

Following a high dose aerosol infection with *M. massiliense* reference strain CIP 108297 and Brazilian outbreak strain CRM-0019 in GKO mice (∼1,000 bacilli per animal), the bacterial loads in the lungs and spleens were quantified on days 1, 30 and 60 after the challenge. Aerosol infection with the outbreak strain *M. massiliense* CRM-0019 resulted in a sustained infection in the lungs and spleens with more than 10^3^ organisms recovered from both organs at 60 days (Figures 2A and 2B). In comparison, GKO mice infected with the reference strain of *M. massiliense* showed complete clearance of this strain in the lungs and spleens by day 60 (Figures 2A and 2B).

### Lung histopathology in GKO mice after aerosol challenge with *Mycobacterium massiliense*

**Figure 3:**
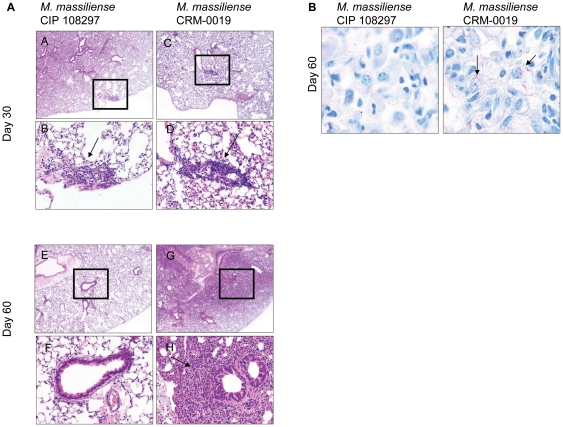
Lung histology from *M. massiliense* infected IFNγ-KO mice. Panel A, A-H shows representative lung histopathology from the *M. massiliense* CIP 108297- and epidemic CRM-0019-infected animals on day 30 and 60 of the infection. Tissue sections are stained with hematoxylin and eosin. The lower photograph depicts the magnified region denoted by the square (upper photograph). CRM-0019-infected mice (panel A: D and H) showed an increased rate of granuloma (arrows) progression and involvement of larger areas of the lung compared to the control mice (panel A: B and E). Panel B shows representative lung acid fast staining bacilli at day 60 after *M. massiliense* CIP 108297 and CRM-0019 infection. Total magnification, panel A [A, C, E, G]  =  10x; panel A [B, D, F, H]  =  20x; panel B  =  100x.

Lung histopathology following high dose aerosol challenge with the reference and outbreak *M. massiliense* strains was characterized in GKO mice (Figure 3). At day 30 following the challenge, when there was bacterial persistence in the lungs for both strains, lung tissues demonstrated a local inflammatory response denoted by peribronchiolar inflammatory infiltrates (Figure 3, panel A, A-D, arrow). However, by day 60, while the reference *M. massiliense* strain showed complete bacterial clearance and healthy lung tissue (Figure 3, panel A, E–F), the lungs of the CRM-0019-infected GKO mice showed large areas of consolidation (Figure 3, panel A, G–H). In addition, CRM-0019-infected mice showed peribronchiolar inflammatory infiltrates with large aggregates of foamy cells and the presence of macrophages with multiple intracellular acid fast staining bacilli (Figure 3B).

### Macrophage and dendritic cell cytokine expression during *Mycobacterium massiliense* infection

**Figure 4:**
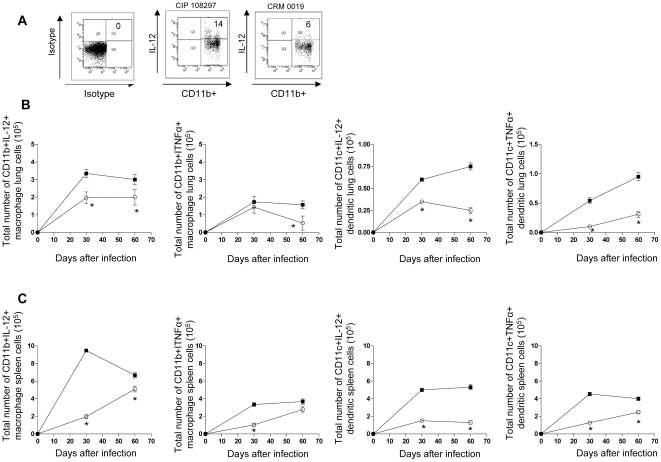
Decreased macrophage and dendritic cell expression of IL-12 and TNFα in the lungs and spleens of IFNγ-KO mice infected with the epidemic strain *M. massiliense* CRM-0019. Mice infected with *M. massiliense* CIP 108297 (closed square) and the epidemic strain *M. massiliense* CRM-0019 (open circle) were assayed by flow cytometry on days 30 and 60. Panel A shows representative dot plots of lung cells from the isotype control, reference CIP 108297 and the outbreak *M. massiliense* CRM-0019 strain after 30 days of infection in mice primarily gated on CD11b+ macrophages cells expression of IL-12. Panels B and C show the total number of macrophage (CD11b+) and dendritic (CD11c+) cells expressing IL-12 or TNFα in the lungs (A) and spleens (B) of both types of infected mice. Results are expressed as the mean number of CD11b^+^IL-12^+^, CD11b^+^ TNFα^ +^,CD11c^+^IL-12^+^, CD11c^+^ TNFα^ +^ (± SEM, n = 5) T cells in the lungs and spleens. Student *t*-test, *p<0.050.

Given the clear differences seen in cell influx indicated by the histological analysis, we conducted a comparative flow cytometric analysis of alveolar macrophages (CD11b+) and dendritic cell (CD11c+) populations from lungs and spleens of *M. massiliense* CRM-0019-infected mice compared to CIP 108297-infected mice. Figure 4 A shows representative dot plots of lung cells from the isotype control, reference CIP 108297 and the outbreak *M. massiliense* CRM-0019 strain after 30 days of infection in mice primarily gated on CD11b+ macrophages cells expression of IL-12. As shown in Figure 4 B and C, macrophages and dendritic cells from the CRM-0019-infected mice showed reduced numbers of lung and spleen IL-12 and TNFα producing macrophage and dendritic cells compared to the reference strain CIP 108297.

### CD4+ and CD8+ T cell cytokine expression during *Mycobacterium massiliense* infection

We next sought to investigate the differences in the CD4+ and CD8+ T cells in the lungs and spleens of mice infected with the epidemic strain *M. massiliense* CRM-0019 compared to reference strain CIP 108297 after 30 and 60 days of infection. We evaluated CD4+ and CD8+ T cells ability to produce the protective cytokine TNFα required for efficient granuloma formation [34]. We also evaluated CD4+ and CD8+ T cells ability to induce immunosuppressive cytokines such as IL-4 which is considered to increase host susceptibility to intracellular pathogens [20].

**Figure 5:**
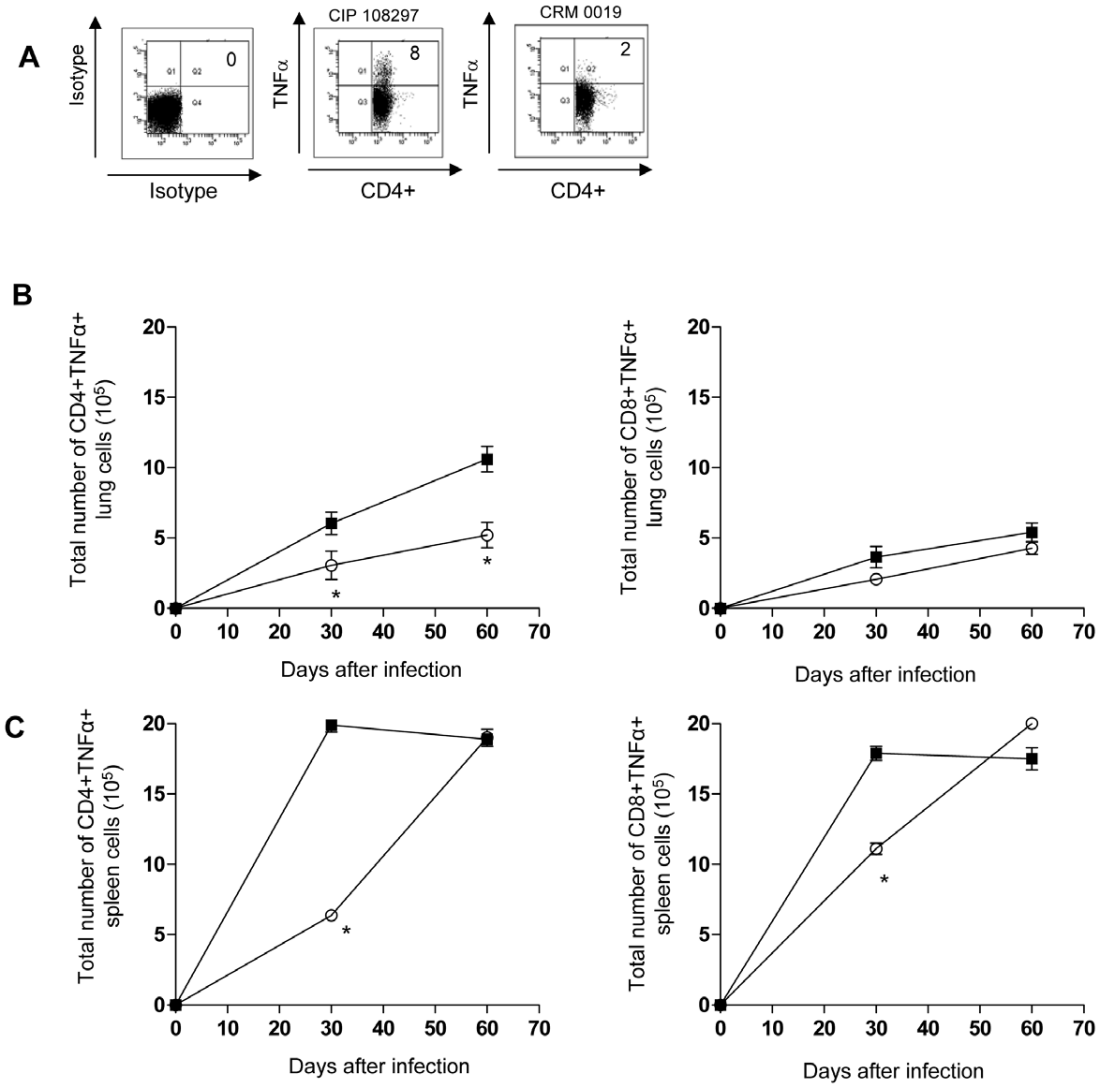
Decreased early CD4+ and CD8+ T cell expression of TNFα in the lungs and spleens of IFNγ-KO mice infected with the epidemic strain *M. massiliense* CRM-0019. Mice infected with an aerosol dose of *M. massiliense* CIP 108297 (closed square) and CRM-0019 (open circle) were assayed by flow cytometry on days 30 and 60. Panel A shows representative dot plots of lung cells from the isotype control, reference CIP 108297 and the outbreak *M. massiliense* CRM-0019 strain after 30 days of infection in mice primarily gated on CD4+ T cells expression of TNFα. Panels B and C show decreased CD4+ and CD8+ T cell expression of TNFα in the lungs (A) and spleens (B) of mice infected with the epidemic strain CRM-0019 compared to CIP 108297 (± SEM, n = 5). Results are expressed as the mean number of CD4^+^TNFα^+^, CD8^+^TNFα^+^, (± SEM, n = 5) T cells in the lungs and spleens. Student *t*-test, *p<0.050.

Figure 5 A shows representative dot plots of lung cells from the isotype control, reference CIP 108297 and the outbreak *M. massiliense* CRM-0019 strain after 30 days of infection in mice primarily gated on CD4+ T cells expression of TNFα. Mice infected with CRM-0019 showed significant reduced numbers of lung and spleen TNFα–expressing CD4+ T cells by day 30 of infection compared to the reference strain (Figure 5 B-C). However, animals infected with the epidemic strain during chronic infection showed similar level of TNFα–expressing CD4+ and CD8+ T cells in the spleen as the reference strain (Figure 5 C). The two types of infected mice did not differ in lung and spleen IL-4–expressing CD4+ and CD8+ T cells (data not shown).

### CD4+Foxp3 regulatory T cell expression during </emph>Mycobacterium massiliense</emph> infection

**Figure 6:**
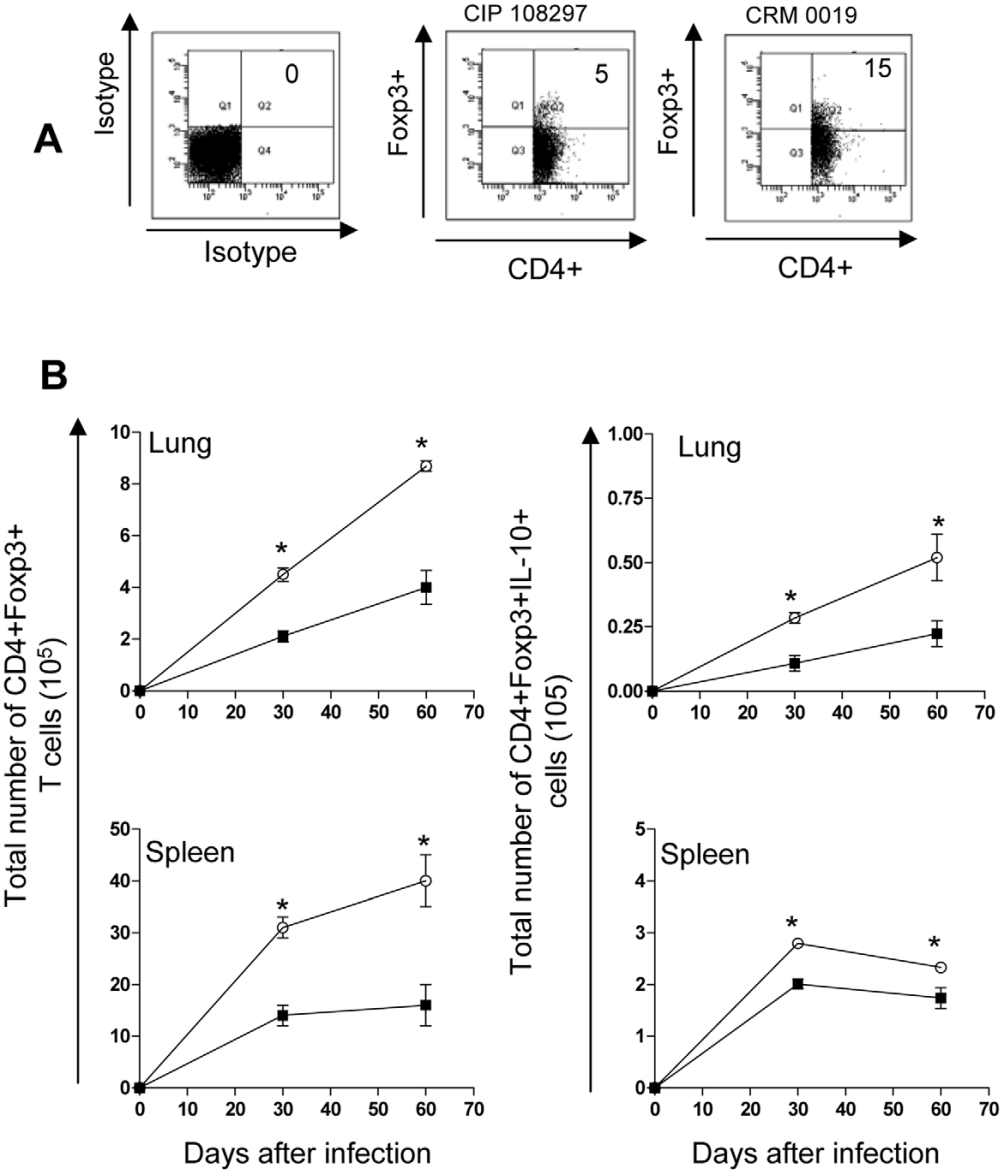
Increased CD4+ Foxp3+IL-10+ regulatory T cell expression in the lungs and spleens of IFNγ-KO mice infected with *M. massiliense* CRM-0019. Lung cells obtained from mice infected with *M. massiliense* CIP 108297 (closed square) and CRM-0019 (open circle) were assayed by multi-parametric flow cytometry on days 30 and 60. Panel A shows representative dot plots of lung cells from the isotype control, reference CIP 108297 and the outbreak *M. massiliense* CRM-0019 strain after 30 days of infection in mice primarily gated on CD4+ T cells expression of Foxp3+. Panel B shows that mice infected with *M. massiliense* CRM-0019 had a significant increase in CD4+Foxp3+ and CD4+Foxp3+ IL-10+ regulatory T cells in the lungs and spleens at 30 and 60 days of infection compared to *M. massiliense* CIP 108297-infected mice. Results are expressed as the mean number of CD4+Foxp3+ and CD4^+^Foxp3+ IL-10+ (± SEM, n = 5) T cells in the lungs and spleens. Student t-test, *p<0.050.

To try to understand these differences seen between the epidemic strain CRM-0019 and the reference M. massiliense strain CIP 108297, we performed studies in which we re-examined the T cell response. Figure 6 A shows representative dot plots of lung cells from the isotype control, reference CIP 108297 and the outbreak *M. massiliense* CRM-0019 strain after 30 days of infection in mice primarily gated on CD4+ T cells expression of Foxp3+. The results in Figure 6 B clearly show an increased number of CD4+Foxp3+IL-10+ T cells in the lungs and spleens of mice infected with the epidemic strain compared to the reference strain at both time points. In fact, compared to CIP 108297-infected mice, those infected with CRM-0019 demonstrated increased numbers of both CD4^+^Foxp3^+^ T cells and CD4^+^Foxp3^+^ IL10+ T cells in the lungs and spleens during the course of infection.

## Discussion

Owing to its relatively recent recognition as a novel *Mycobacterium* species [2], not much is known about the virulence and pathogenicity of *M. massiliense*. The limited information presently available suggests that the clinical manifestations of the diseases caused by *M. massiliense* in humans are similar to those caused by *M. abscessus* [14]. The results of this study clearly show that *M. massiliense* clinical isolates with different genetic backgrounds can significantly differ in their ability to replicate and persist inside macrophages and induce distinct immune responses contributing to the different virulence phenotypes in mice. The Brazilian epidemic BRA100 strain *M. massiliense* CRM-0019 clearly replicates more efficiently than the reference strain CIP 108297 in mouse bone marrow macrophages resulting in rapid cell lysis. It is also more virulent for GKO mice than is strain CIP 108297, persisting in the lungs and spleens and inducing more severe lung and spleen pathology. Mice infected with strain CIP 108297 eventually clear the infection showing an absence of residual bacilli and organ pathology 60 days post-infection. Several reasons may account for the enhanced persistence of CRM-0019 *in vivo* including the increased ability of this isolate to replicate and survive inside macrophages and, potentially, our novel observation here that this particular *M. massiliense* isolate expands a regulatory T cell response that attempts to dampen the progression of organ pathology associated with bacterial persistence but also suppresses the protective TNFα and IL-12 [TH1] response required for mycobacterial containment and eventual elimination.

In this regard, we found evidence that immunodeficient GKO mice administered the epidemic BRA100 strain *M. massiliense* CRM-0019 through the aerosol route expand a weak TH1 response, compared to the reference strain CIP 108297. Recent studies have shown that immunocompetent C57BL/6 and BALB/c mice infected with a large intravenous dose of a *M. massiliense* BRA100 isolate closely related to CRM-0019 developed a robust TH1 response consisting of macrophage, dendritic cell and natural killer cell activation, induced by IL-12 and IL-17 [35]. However, there are important differences between their study and ours which raise new interesting questions. First, they intravenously infected their immunocompetent mice with a very large inoculum of *M. massiliense* CRM-0019 (1×10^6^ organisms per mice, with concentration level reaching 10^7^ CFU in the liver, spleen and lungs [35]. Thus, although their murine model of intravenous infection revealed important immunological findings, it may not reflect the typical bacterial numbers nor immune responses elicited by immunodeficient humans during a nosocomial, wound or aerosol infection [7], [21]. Prior studies have shown that increased doses of NTM can induce increasingly robust immune responses [20]. Second, an intravenous infection which kinetically spreads to all organs rapidly causing rapid bacterial dissemination does not reflect bacilli deposited in soft tissue during a surgical procedure nor a pulmonary infection [36]. Our model was developed to use a dose of bacteria and an immunodeficient animal model which best reflects those individuals which generally become infected with such opportunistic pathogens as those from the *M. abscessus*-*M. massiliense* group.

The apparent lack of TH1 immunity during aerosol infection with the outbreak strain *M. massiliense* CRM-0019 was due to reduced numbers of CD11b^+^ cells producing TNF-α and IL-12 cells at the site of infection. In addition, CD11c+ dendritic cells were also expressing less TNF-α and IL-12. Importantly, in our comparative evaluation of T cell responses in *M. massiliense* CRM-0019 and CIP 108297-infected mice, we observed reduced numbers of CD4+ T cells secreting TNFα in the lungs and spleens of CRM-0019-infected mice at 30 days, while at the same time increased numbers of CD4+Foxp3+IL-10^+^ CD4 cells was observed. This is the first observation that non-tuberculous rapidly-growing mycobacteria are capable of inducing the development of immunosuppressive regulatory T cells. Earlier reports have shown that clinical isolates of *M. tuberculosis* vary in their ability to induce regulatory T cells with possible consequences on virulence and host protective immunity [23], [37]. Moreover, there is evidence that patients with active tuberculosis have raised levels of circulating regulatory T cells [38], [39]. Thus, regulatory T cell induction may be inherent in mycobacterial host immunity and not just responding to the acute lung damage caused by highly virulent *M. tuberculosis*. Our study suggests that IL-10, a product of regulatory T cells as well as macrophages, may play a role in maintaining the stability of persistent NTM disease as has also been suggested in the case of *Bordetella pertussis* infection [40].

A likely correlate of our important findings is that the ability of some *M. massiliense* isolates and, perhaps, closely related *Mycobacterium species*, to persist *in vivo* through increased resistance to the bactericidal mechanisms of macrophages and the modulation of T regulatory cells may account, at least in part, for the extreme difficulty of treating the infections they cause [7] and the lack of correlation between their susceptibility to drugs under axenic conditions and *in vivo*.

At present the only available vaccine against tuberculosis, *M. bovis* Bacillus Calmette-Guérin (BCG), has proven unreliable in being able to protect against pulmonary tuberculosis in adults [41]. It has been proposed by some epidemiological studies that BCG protects the host in countries where low levels of environmental NTM exposure occurs compared to lack of BCG protection in developing countries where high levels of environmental NTM exposure occurs [42]. We propose that the widely varying degrees of BCG efficacy present in different studies may be due to environmental NTM exposure and pre-sensitization of individuals to regulatory T cell expansion resulting in cross reactivity during *M. tuberculosis* [24] disease.

Further research is required to link the virulence and molecular mechanisms underlying the increased pathogenicity of BRA100 *M. massiliense* epidemic isolates, whether related to changes in surface composition under the selective pressure of GTA disinfection or otherwise, which are presently being investigated in our laboratories.

## Materials and Methods

### Bacterial Cultures

CIP 108297 (the *M. massiliense* type strain) [2] and the BRA100 isolate, CRM-0019 [13], were routinely grown at 30°C in liquid Middlebrook 7H9-OADC medium (Difco) supplemented with 0.05% Tween 80 or on solid Middlebrook 7H11-OADC medium.

### GTA-susceptibility testing

The susceptibility of *M. massiliense* to glutaraldehyde in suspension tests was determined as described by Griffiths *et al*. [43].

### Mice

Specific-pathogen-free female GKO mice, from 6 to 8 weeks old, were purchased from the Jackson Laboratories, Bar Harbor, Maine. Mice were maintained in the Biosafety Level III animal laboratory at Colorado State University, and were given sterile water, mouse chow, bedding, and enrichment for the duration of the experiments. The specific pathogen-free nature of the mouse colonies was demonstrated by testing sentinel animals. All experimental protocols were approved by the Animal Care and Usage Committee of Colorado State University. The CSU animal assurance welfare number is A3572-01.

### Experimental infection of BMDM and bacterial enumeration

Macrophages were infected with mycobacteria, and 2 hours later, the monolayers were washed to remove extracellular bacilli. The numbers of intracellular mycobacteria were measured by plating. Briefly, monolayers were washed at each time point to remove extracellular bacilli and 1 ml double-distilled H_2_O containing 0.05% Tween 80 was added to monolayers and incubated for 10 min to lyse macrophages. After passing through a 26-gauge needle five times, the lysates were serially diluted and plated onto Middlebrook 7H11 agar plates. Monolayers that weren't lysed were replenished with fresh medium.

### Cellular viability assays

BMDM cell death was assayed by trypan blue exclusion and determined by flow cytometry using BD™ Viability Counting Beads, as described by the manufacturer (BD PharMingen, San Jose, CA USA 95131) cell viability-staining methods [32].

### Experimental infections in mice

Mice were challenged with reference strain Mycobacterium massiliense CIP 108297 and a clinical epidemic BRA100 strain, Mycobacterium massiliense CRM-0019, using a Glas-Col (Terre Haute, Inc.) aerosol generator calibrated to deliver either a HDA of ∼1,000 bacilli per animal. At days 1, 30 and 60 following infection, bacterial loads in the lungs and spleen, lung histology, and mononuclear and lymphocytic cellular expressions were determined. Bacterial counts were determined by plating serial dilutions of homogenates of lungs on nutrient 7H11 agar and counting colony-forming units after 5-10 days incubation at 30°C. A total of five animals were infected for each time point.

### Histological analysis

The accessory lobe of the lung from each mouse was fixed with 10% formalin in phosphate buffered saline (PBS). Tissue sections were stained using haematoxylin and eosin and acid-fast stains as previously reported [20], [23].

### Lung cell digestion

Briefly, single cell suspensions were prepared as described previously [20]. Lungs and spleens were aseptically removed, teased apart and treated with a solution of deoxyribonuclease IV (DNAse) (Sigma Chemical, 30 µg/ml) and collagenase XI (Sigma Chemical, 0.7 mg/ml) for 45 min at 37°C. To obtain a single-cell suspension, the organs were gently passed through cell strainers (Becton Dickinson, Lincoln Park, NJ). The remaining erythrocytes were lysed with Gey's solution (0.15 M NH_4_Cl, 10 mM KHCO_3_) and the cells were washed with Dulbecco's modified Eagle's minimal essential medium. Cell suspensions from each individual mouse were incubated with monoclonal antibodies labeled with fluorescein isothiocyanate (FITC), phycoerythrin (PE), peridinin chlorophyll-a protein (PerCP), or allophycocyanin (APC) at 4°C for 30 minutes in the dark as described previously [20]. Total cell numbers were determined by flow cytometry using BD™ Liquid Counting Beads, as described by the manufacturer (BD PharMingen, San Jose, CA USA 95131). All analyses were performed with an acquisition of at least 100,000 total events.

### Intracytoplasmic cytokine staining

Cells were first stained for cell surface markers as indicated above and thereafter the same cell suspensions were prepared for intracellular staining as described before [20], [23]. For flow cytometry analysis, single-cell suspensions of lung from each mice were re-suspended in PBS (Sigma-Aldrich) containing 0.1% of Sodium Azide (PBS+Na/Az). Cells were incubated in the dark for 25 min at 37°C with pre-determined optimal titrations of specific antibody (directly conjugated to fluorescein isothiocyanate (FITC), phycoerythrin (PE), peridin-cholorophyll-protein (PerCP), allophycocyanin (APC), Pacific Blue, Alexa 700); or after biotin antibody incubations washed and incubated for 25 minutes more with streptavidin Qdot800 (Invitrogen), followed by two washes in PBS containing 4% sodium azide. Measurement of intracellular cytokines was conducted by pre-incubating lung cells with monensin (3 µM) (Golgi Stop, BD PharMingen), anti-CD3 and anti-CD28 (both at 0.2 µg/10^6^ cells) for 4 h at 37°C, 5% CO_2_. The cells were then surface stained, incubated for 30 minutes at 37°C, washed then fixed and permeabilized with Perm Fix/Perm Wash (BD Pharmingen). Finally, the cells were stained with intracellular Foxp3 (FJK-16s), IL-12 (clone REM-11) TNFα (clone TB 15), or its respective isotype controls (BD Pharmingen) for a further 30 min. All the samples were run on a Becton Dickinson LSR-II and data were analyzed using FACSDiva v5.0.1 software. Cells were gated on lymphocytes based on characteristic forward and side scatter profiles. Individual cell populations were identified according to their presence of specific fluorescent-labeled antibodies. All the analyses were performed with a minimum acquisition of 100,000 events. Our laboratory routinely uses the Becton-Dickinson Company LSR II flow cytometer fluorescence minus one (FMO) technology comparing the isotype control verses the fluorescence of the subpopulations evaluated as an internal quality control in addition to the standard isotype verses isotype control. We have verified that no significant differences exist in values between our isotype verse isotype control compared to our isotype verses the fluorescence of the subpopulations evaluated in our study.

### Statistical analysis

Data are presented using the mean values from 5 mice per group and from values from replicate samples and duplicate or triplicate assays. The Student t-test test was used to assess statistical significance between groups of mice.

## Supporting Information

Information S1 **(A) Partial sequencing of the** ***hsp65*** **and** ***rpoB*** **genes of** ***M. massiliense*** **CIP 108297 and CRM-0019.** The corresponding sequences of *M. abscessus* ATCC 19977 are included in the alignments as a reference. PCR amplification were performed as described by Ringuet *et al.* (1999) and Kirshner *et al*. (1993) (see references below). **(B) Growth rates of** ***M. massiliense*** **CIP 108297 and CRM-0019 in 7H9-OADC-Tween 80 broth at 30°C.** *M. massiliense* CIP 108297 (diamonds); *M. massiliense* CRM-0019 (rectangles). **(C) GTA susceptibility of** ***M. massiliense*** **CIP 108297 and CRM-0019.** Results are expressed as CFU counts upon exposure of the test organisms to 2.2% GTA (under the formulated form of Cidex®, Johnson & Johnson) for 0 to 30 min. *M. massiliense* CIP 108297 (diamonds); *M. massiliense* CRM-0019 (rectangles).
(PDF)
