# Supplementary material for: Increased Virulence of an Epidemic Strain of Mycobacterium massiliense in Mice
Source: PLoS One. 2011 Sep 12;6(9):e24726. doi: 10.1371/journal.pone.0024726 (PMC3171484; doi:10.1371/journal.pone.0024726)

Supporting Information S1

(A) Partial sequencing of the *hsp65* and *rpoB* genes of *M. massiliense* CIP 108297 and CRM-0019. The corresponding sequences of *M. abscessus* ATCC 19977 are included in the alignments as a reference. PCR amplification were performed as described by Ringuet *et al*. (1999) and Kirshner *et al*. (1993) (see references below).

hsp65

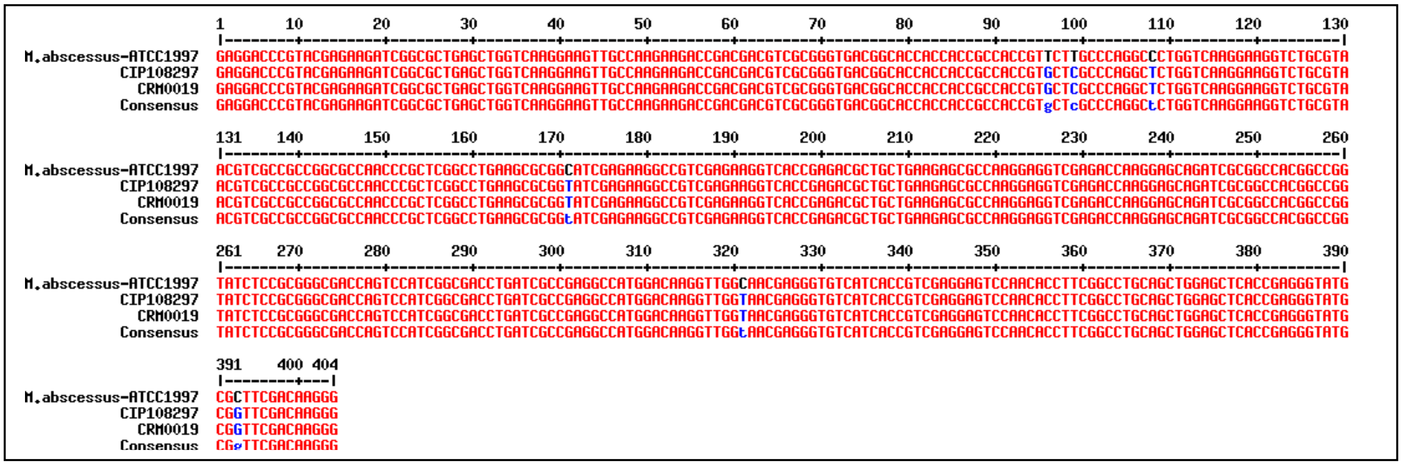

rpoB

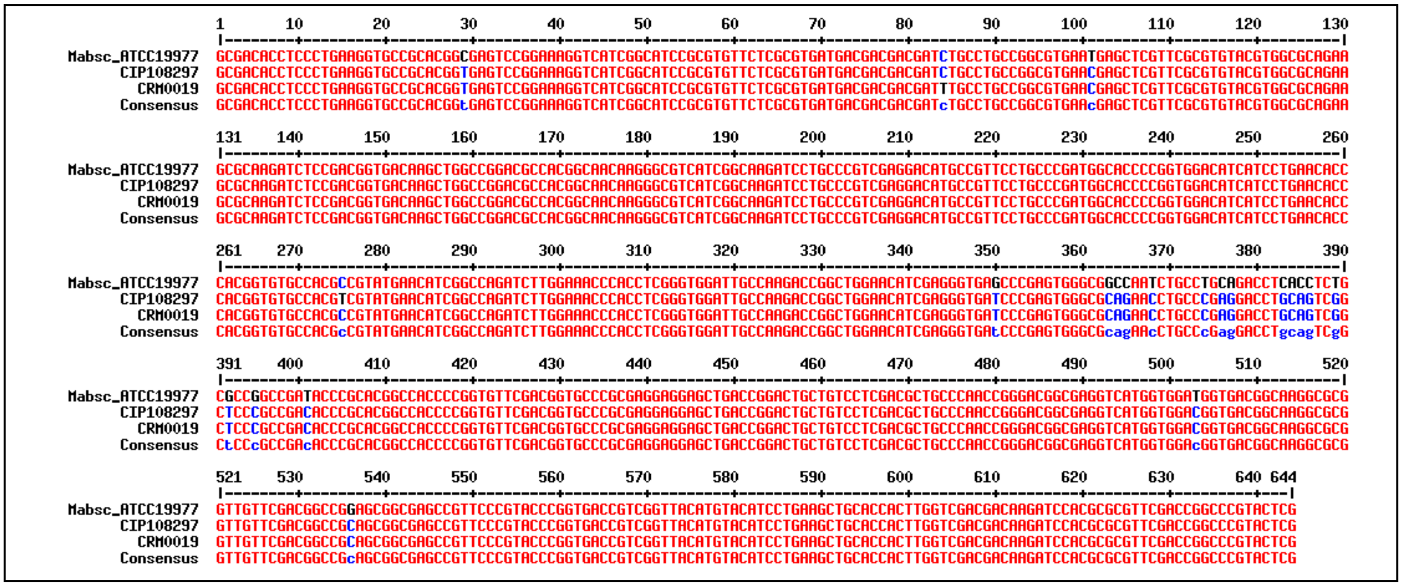

Kirschner P, Springer B, Vogel U., Meier A., Wrede A., Kiekenbeck M., Bange FC, Bottger EC. (1993) Genotypic identification of mycobacteria by nucleic acid sequence determination: Report of a 2-year experience in a clinical laboratory. J. Clin. Microbiol. 31:2882-2889.

Ringuet H, Akoua-Koffi C, Honore S, Varnerot A, Vincent V, Berche P, Gaillard JL, Pierre-Audigier C (1999) *hsp65* sequencing for identification of rapidly growing mycobacteria. J. Clin. Microbiol. 37:852-857.

(B) Growth rates of *M. massiliense* CIP 108297 and CRM-0019 in 7H9-OADC-Tween 80 broth at 30ºC.

M. massiliense CIP 108297 (diamonds); *M. massiliense* CRM-0019 (rectangles).

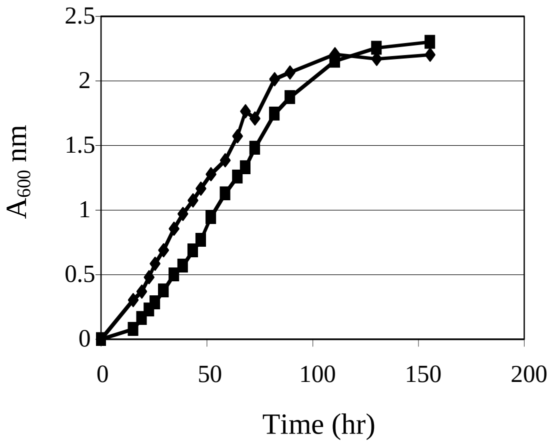

(C) GTA susceptibility of *M. massiliense* CIP 108297 and CRM-0019.

Results are expressed as CFU counts upon exposure of the test organisms to 2.2% GTA (under the formulated form of Cidex®, Johnson & Johnson) for 0 to 30 min. *M. massiliense* CIP 108297 (diamonds); *M. massiliense* CRM-0019 (rectangles).

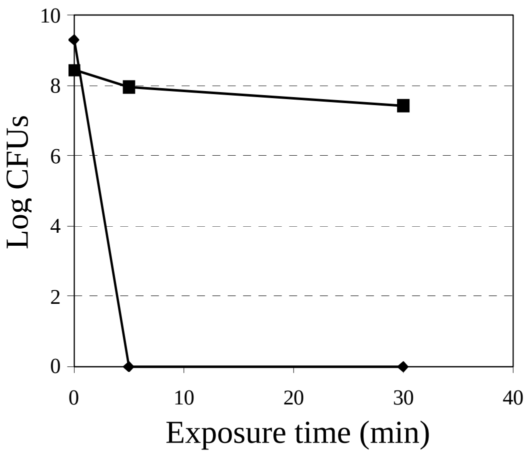

Supplement: Information S1 — (A) Partial sequencing of the hsp65 and rpoB genes of M. massiliense CIP 108297 and CRM-0019. The corresponding sequences of M. abscessus ATCC 19977 are included in the alignments as a reference. PCR amplification were performed as described by Ringuet et al. (1999) and Kirshner et al. (1993) (see references below). (B) Growth rates of M. massiliense CIP 108297 and CRM-0019 in 7H9-OADC-Tween 80 broth at 30°C. M. massiliense CIP 108297 (diamonds); M. massiliense CRM-0019 (rectangles). (C) GTA susceptibility of M. massiliense CIP 108297 and CRM-0019. Results are expressed as CFU counts upon exposure of the test organisms to 2.2% GTA (under the formulated form of Cidex®, Johnson & Johnson) for 0 to 30 min. M. massiliense CIP 108297 (diamonds); M. massiliense CRM-0019 (rectangles). (PDF) [file pone.0024726.s001.pdf]
